# Supplementary material for: The Role of Cilostazol, a Phosphodiesterase-3 Inhibitor, in the Development of Atherosclerosis and Vascular Biology: A Review with Meta-Analysis
Source: Int J Mol Sci. 2024 Feb 23;25(5):2593. doi: 10.3390/ijms25052593 (PMC10932209; doi:10.3390/ijms25052593)
Supplement: Supplementary file 1 [file ijms-25-02593-s001.zip › ijms-2843438-supplementary.pdf]

Supplementary Table S1. Search strategy in detail.

Category	Search terms
Terms	Pubmed: MeSH term<br>Embase: Entrée term with synonyms on major focus<br>Cochrane: Suggested keywords
Intervention/Comparison	Pubmed: cilostazol<br>Embase: cilostazol<br>Cochrane: cilostazol
Outcomes	Pubmed: 'major adverse cardiovascular events' OR 'myocardial infarction' OR 'stroke' OR 'cardiovascular diseases'<br>Embase: 'cardiovascular disease' OR 'heart infarction' OR 'cerebrovascular accident'<br>Cochrane: 'cardiovascular event rate' OR 'cardiovascular disease' OR 'myocardial infarction' OR stroke
Study design	Randomized controlled trial
Intervention time	At least 3 months<br>Studies with intervention periods of less than 3 months are excluded, as these cases often transitioned to other conventional therapies or targeted secondary prevention that could be attributed to factors unrelated to atherosclerosis.
Restrictions	Filter: humans, English, randomized controlled trial<br>Search field: title or abstract<br>Publication type: article<br>Not animals
Search date	From 2000/1/1 to 2023/7/13
Search results	Pubmed: 83 results<br>Embase: 188 results<br>Cochrane: 225 results

Supplementary Table S2. Definition of cardiovascular events.

Trial	Major adverse cardiovascular events
Gotoh F et al. 2000 [95]	Cerebral infarction, intracranial hemorrhage, MI, or vascular death
Douglas JS et al. 2005 [96]	Major adverse cardiovascular events not specified
Chen YD et al. 2006 [97]	Death, nonfatal MI, stent thrombosis, TLR, or stroke
Lee SW et al. 2007 [98]	Death, MI, or target vessel revascularization
Hiatt WR et al. 2008 [99]	Total number of cerebrovascular accidents, carotid artery stenosis, femoral artery occlusion, and cardiac arrest
Huang Y et al. 2008 [100]	Recurrent stroke, new MI, transient ischemic attack, vascular event—including pulmonary embolism, deep venous thrombosis, or peripheral arterial occlusion disorder—death from vascular causes, or death from any other cause
Lee SW et al. 2008 [101]	Death, MI, or target lesion revascularization
Guo JJ et al. 2009 [102]	Total number of deaths, acute coronary event, intracerebral hemorrhage, and ischemic stroke
Han Y et al. 2009 [103]	Cardiac death, nonfatal MI, stroke, or TVR

Soga Y et al. 2009 [104]	Total number of deaths, nonfatal MI, stroke, and repeat revascularization
Katakami N et al. 2010 [18]	Sudden cardiovascular death, new onset or recurrence of cerebral infarction or transient cerebral ischemia, development of angina or acute myocardial ischemia, or exacerbation of peripheral artery disease
Shinohara Y et al. 2010 [20]	Any stroke, transient ischemic attack, angina pectoris, MI, heart failure, or any hemorrhage requiring hospital admission
Kwon SU et al. 2011 [105]	Vascular death, nonfatal stroke, or nonfatal MI
Lee SW et al. 2011 [106]	Death, MI, or ischemic-driven TLR
Lee YS et al. 2011 [107]	Vascular death, nonfatal stroke, nonfatal MI, hospitalization for cardiovascular events
Suh JW et al. 2011 [108]	Cardiac death, MI, ischemic stroke, or TLR
Iida O et al. 2013 [109]	All-cause death, MI, or stroke
Gao W et al. 2013 [110]	Death, MI, or TLR
Youn YJ et al. 2014 [111]	All-cause mortality, any MI, or repeat revascularization
Uchiyama S et al. 2015 [112]	Death, ischemic stroke, myocardial infarct, or other vascular events
Ueda H et al. 2016 [113]	All-cause death, nonfatal MI, nonfatal stroke, or coronary or cerebrovascular revascularization
Zheng X-T et al. 2016 [114]	Cardiac death, MI, or TLR
Tang Y-D et al. 2018 [115]	All-cause death, MI, target vessel revascularization, or stroke
Lee CH et al. 2018 [116]	All-cause death, MI, ischemic stroke, or ischemic-driven TVR
Kim BJ et al. 2018 [117]	Stroke (including hemorrhagic stroke), MI, or vascular death
Hong S et al. 2019 [118]	Acute MI, coronary-artery bypass graft, PCI, ischemic stroke, hemorrhagic stroke, or death due to any cardiovascular event
Toyoda K et al. 2019 [119]	Vascular death, stroke, or MI
Chen Y-C et al. 2019 [120]	Death, MI, or revascularization of the original lesion
Uchiyama S et al. 2021 [121]	Stroke, MI, or vascular death
Kalantzi K et al. 2021 [122]	Acute ischemic stroke, transient ischemic attack, acute MI, or death from vascular causes
Sohn M et al. 2022 [123]	Cardiovascular death, nonfatal MI, nonfatal stroke, angina, or hospitalization for heart failure
Lin J-L et al. 2022 [124]	Cardiovascular death, nonfatal MI, nonfatal stroke, hospitalization for heart failure, or unplanned coronary revascularization
Wardlaw JM et al. 2023 [125]	Total number of stroke, transient ischemic attack, MI, or all-cause death
Park S et al. 2023 [129]	All-cause death, recurrent MI, stroke, and repeat revascularization

MI, myocardial infarction; PCI, percutaneous coronary intervention; TLR, target lesion revascularization; and TVR, target vessel revascularization

Study	D1: Bias arising from the randomization process.	D2: Bias due to deviations from intended intervention.	D3: Bias due to missing outcome data.	D4: Bias in measurement of the outcome.	D5: Bias in selection of the reported result.
Gotoh F et al. 2000	+	?	+	?	+
Douglas JS et al. 2005	+	+	?	+	+
Chen YD et al. 2006	+	+	+	+	+
Lee SW et al. 2007	+	+	+	+	+
Hiatt WR et al. 2008	?	+	+	+	+
Huang Y et al. 2008	+	+	+	?	+
Lee SW et al. 2008	+	+	+	+	+
GUO JJ et al. 2009	+	+	?	+	?
Han Y et al. 2009	+	+	+	+	+
Soga Y et al. 2009	+	+	+	+	+
Katakami N et al. 2010	+	+	+	+	+
Shinohara Y et al. 2010	+	+	+	+	+
Kwon SU et al. 2011	+	+	+	+	?
Lee SW et al. 2011	+	+	+	+	+
Lee YS et al. 2011	+	+	+	+	+
Suh JW et al. 2011	+	?	+	+	+
Iida O et al. 2013	+	+	?	+	+
Gao W et al. 2013	+	+	+	+	+
Youn YJ et al. 2014	+	+	+	+	+
Uchiyama S et al. 2015	+	+	+	+	+
Ueda H et al. 2016	+	+	+	+	+
Zheng X-T et al. 2016	+	?	+	?	+
Tang Y-D et al. 2017	+	?	+	+	+
Lee CH et al. 2018	+	+	+	+	+
Kim BJ et al. 2018	+	+	+	+	+
Hong S et al. 2019	+	+	?	+	+
Toyoda K et al. 2019	+	+	?	+	+
Chen Y-C et al. 2019	+	+	+	+	+
Uchiyama S et al. 2021	+	?	+	+	+
Kalantzi K et al. 2021	+	?	+	+	+
Sohn M et al. 2022	+	+	+	+	+
Lin I-L et al. 2022	+	+	+	+	+
Wardlaw JM et al. 2023	+	+	+	+	+
Park S et al. 2023	+	+	+	+	+

Judgement: + low risk; ? some concerns; –high risk

Supplementary Figure S1. Risk-of-bias among trials included in the meta-analysis.

(A)

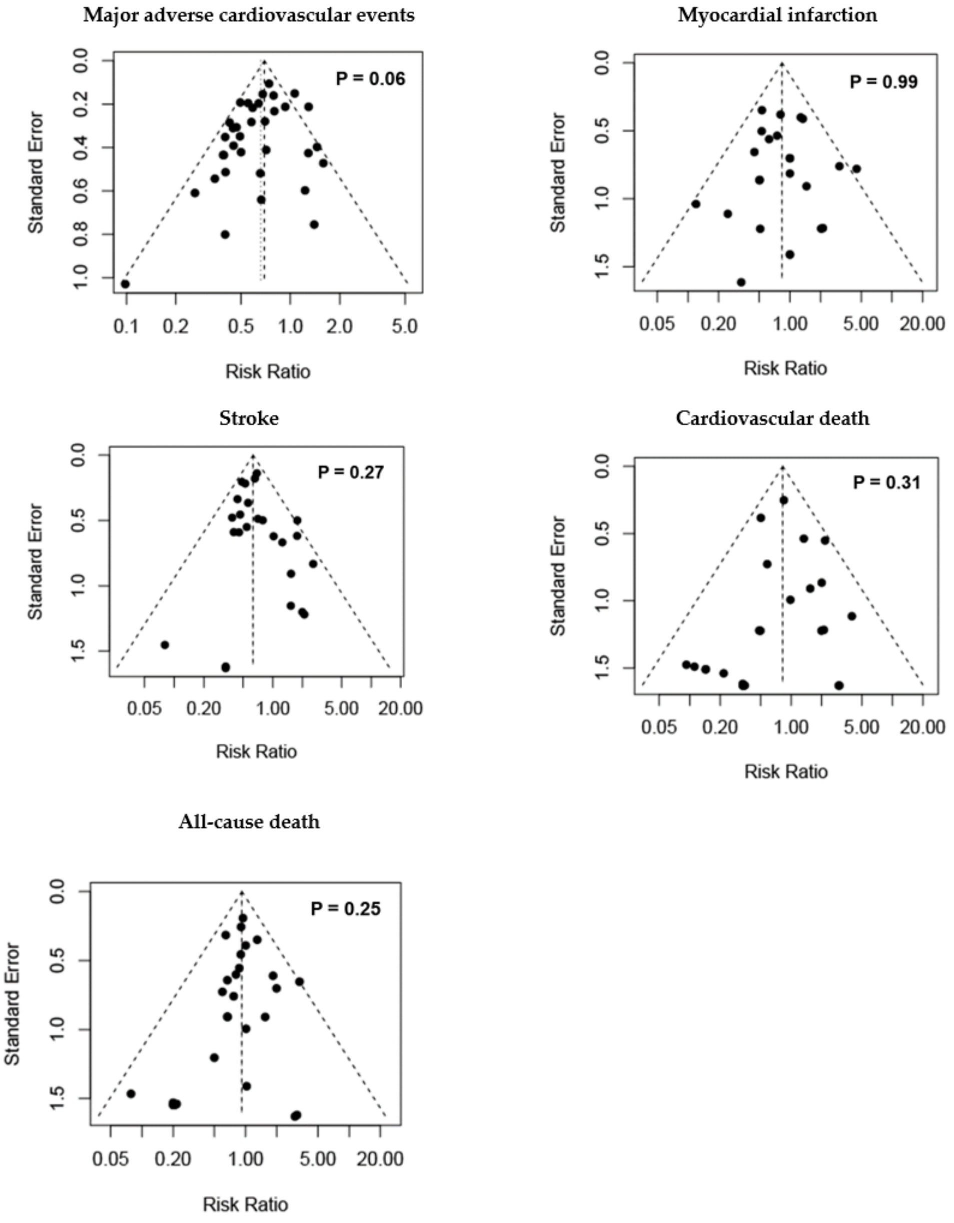

(B)

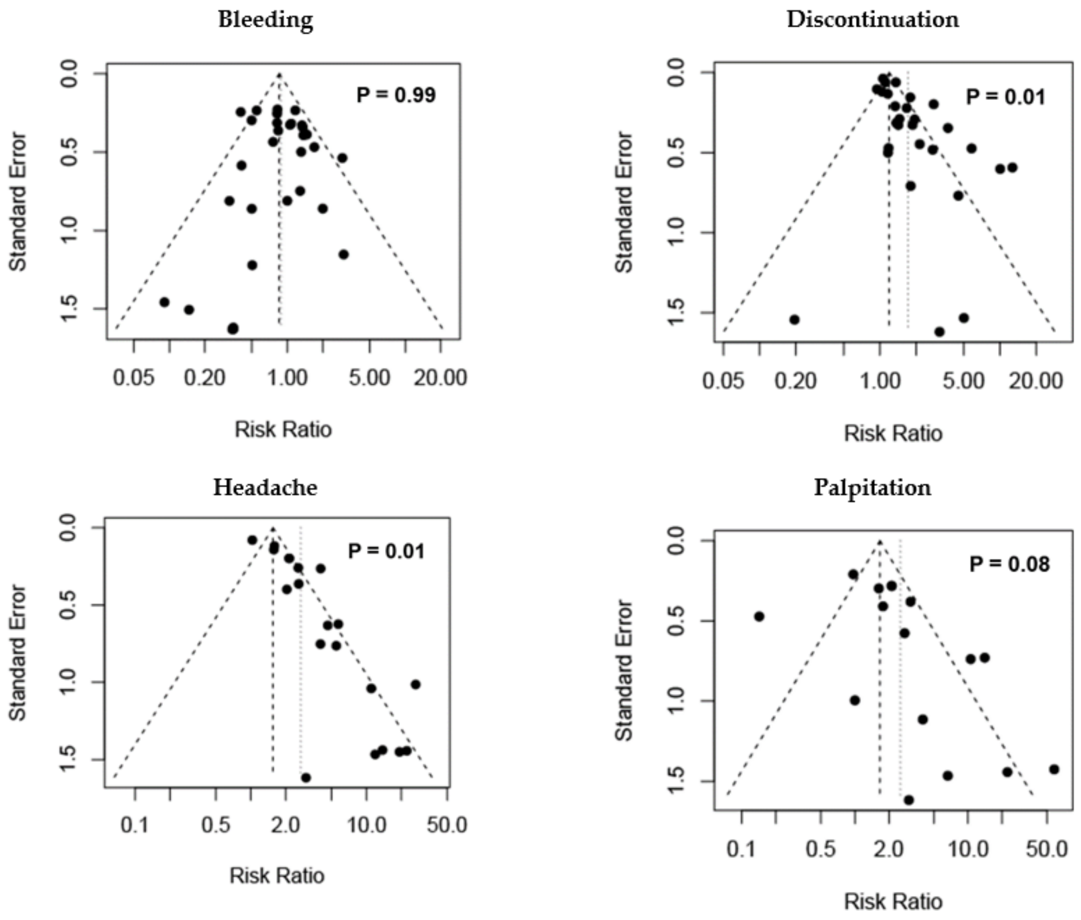

Supplementary Figure S2. Funnel plot of meta-analysis of clinical trials with (A) cardiovascular events and (B) other safety events.

(A)

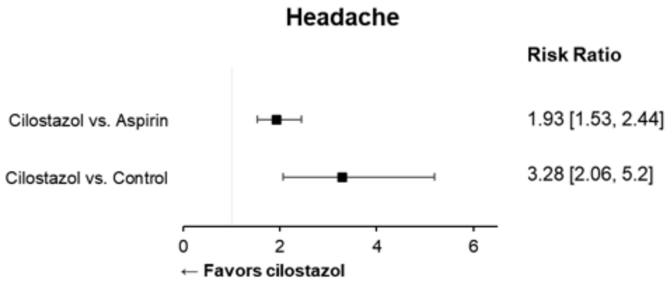

(B)

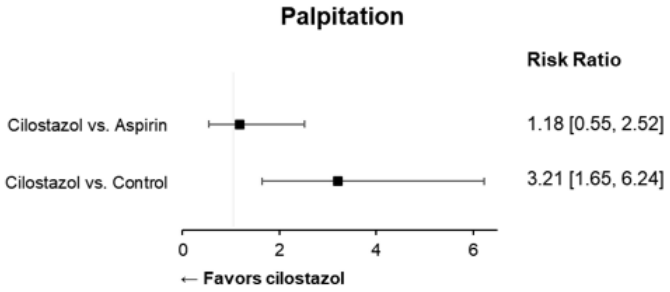

Supplementary Figure S3. Forest plots of cilostazol in adverse events: (A) headache and (B) palpitation.
